# Supplementary material for: The genome sequence of the Violet Copper, Helleia helle (Denis & Schiffermüller), 1776 (Lepidoptera: Lycaenidae)
Source: Wellcome Open Res. 2025 Aug 11;10:429. [Version 1] doi: 10.12688/wellcomeopenres.24699.1 (PMC12432499; doi:10.12688/wellcomeopenres.24699.1)
Supplement: Peer review history

# Reviewer report: Reviewer response for version 1

**DOI:** 10.21956/wellcomeopenres.27215.r129655

**Published:** 2025-09-12

**Authors:** Arun Arumugaperumal

**Affiliations:** 1 Department of Biotechnology, Rajalakshmi Engineering College, Thandalam 602105, Chennai, Tamil Nadu, India

The authors have presented the genome sequencing of a butterfly species *Helleia helle*. This is the second high-quality genome of the organism to be sequenced. The assembly provided is of size 540.27 Mb. The genome assembly consists of 24 chromosomes. Pladevall *et al* had already sequenced the genome of the same organism but the specimen used was a female and the assembly consisted of 25 chromosomes including Z and W chromosomes [1]. This data note has used a male specimen. The genome assembly in both the cases is nearly of the same quality. The genome was sequenced using high-end platforms and assembled using standard procedures. However, the previously published article was not cited. The authors should include the Pladvell *et al* paper in the reference. The voucher photograph in figure 1 is very clear but lacking the morphological details of the organism. Also, the organism is having confusion in name. So it is advisable to include a photograph of alive butterfly as an inset to figure 1. It will help the readers to recognize the species immediately without any confusion.

Are sufficient details of methods and materials provided to allow replication by others?

Yes

Is the rationale for creating the dataset(s) clearly described?

Yes

Are the datasets clearly presented in a useable and accessible format?

Yes

Are the protocols appropriate and is the work technically sound?

Yes

Reviewer Expertise:

Genomics; Bioinformatics

I confirm that I have read this submission and believe that I have an appropriate level of expertise to confirm that it is of an acceptable scientific standard, however I have significant reservations, as outlined above.

## References

[1] : The genome sequence of the Violet Copper, Lycaena helle (Denis & Schiffermüller, 1775). *F1000Research* .2025;14: 10.12688/f1000research.156485.1 10.12688/f1000research.156485.1 40534729 PMC12174908

# Reviewer report: Reviewer response for version 1

**DOI:** 10.21956/wellcomeopenres.27215.r129650

**Published:** 2025-08-30

**Authors:** Laurence Despres

**Affiliations:** 1 Université Grenoble Alpes, Laboratory of Alpine Ecology (Ringgold ID: 56837), Grenoble, Auvergne-Rhône-Alpes, France; 2 Universite Savoie Mont Blanc, Chambéry, France

The authors report on the chromosome-level genome assembly of a male specimen of the violet copper Lycaena (Helleia) helle. The genome assembly is of good quality and was constructed using appropriate methods : Pac Bio HiFi long reads and Hi-C data to confirm scaffolding, following the high standard of the Darwin Tree of Life Project for results presentation.

 I have two main concerns regarding this data note.

 First, the taxonomy used : Helleia helle (Denis & Schiffermüller, 1776), is not the traditionally used taxonomy for the violet copper : Lycaena helle (Denis & Schiffermüller, 1775)-the type species being *Papilio helle* Denis & Schiffermüller, 1775. All the cited references are about Lycaena helle (not Helleia helle). The splitting of the Lycaeninae and Lycaena genus into several genera including the monotypic genus Helleia has been recently proposed (Zhang et al 2020 https://pmc.ncbi.nlm.nih.gov/articles/PMC8794283/#S10 ) based on a phylogeny of the Lycaeninae sub-family using protein coding genes which involved a unique specimen of L. helle from Siberia, but its phylogenetic placement was inconsistent with the phylogeny obtained based on the Z chromosome (Zhang et al. 2022 https://pmc.ncbi.nlm.nih.gov/articles/PMC9645532/ ). This new taxonomy is far from being accepted/known by the lepidopterists’ community and I suggest either to keep the name Lycaena helle or explain the new genus name ‘Helleia (Verity 1947)’ and cite Zhang et al 2020, 2022.

 Second, there is another chromosome level assembly of a female genome of Lycaena helle https://pmc.ncbi.nlm.nih.gov/articles/PMC12174908/ released in January 2025 which should be cited. The present genome is an assembly of a male from Switzerland, while the female genome is from Andorra, and together with the specimen from Siberia, the three genomic datasets could shed light on the taxonomic status of Lycaena/Hellia helle species and its placement in the phylogeny of Lycaeninae.

Are sufficient details of methods and materials provided to allow replication by others?

Yes

Is the rationale for creating the dataset(s) clearly described?

Partly

Are the datasets clearly presented in a useable and accessible format?

Partly

Are the protocols appropriate and is the work technically sound?

Yes

Reviewer Expertise:

evolutionary biology

I confirm that I have read this submission and believe that I have an appropriate level of expertise to confirm that it is of an acceptable scientific standard, however I have significant reservations, as outlined above.

# Reviewer report: Reviewer response for version 1

**DOI:** 10.21956/wellcomeopenres.27215.r129657

**Published:** 2025-08-28

**Authors:** Bin Zhang

**Affiliations:** 1 Qingdao Agricultural University, Qingdao, China

This Data Note presents a high-quality, haplotype-resolved chromosome-level genome assembly of the endangered butterfly Helleia helle (Violet Copper), including PacBio HiFi (17.3 Gb) and Hi-C (85.48 Gb) sequencing data. Key achievements include: Assembly of two haplotypes (540.27/540.28 Mb), with 99.66% of haplotype 1 scaffolded into 24 chromosomes (including Z sex chromosome). BUSCO completeness of 98.4% (lepidoptera_odb10), QV > 64, and mitochondrial genome (15.51 kb). Contextualization of the genome’s utility for conservation genomics of this IUCN-endangered species. I would like to give the decision with “Accept after minor revisions”.

 1. Table 3: State that chromosomes are ordered by size (match Fig 2’s Hi-C map). 

 2. Table 4: Decode "7.7.Q64" (e.g., "Scaffold N50 = 7.7 Mb, QV = 64") and benchmark "6.C.Q40" ("C" = chromosome-level). 

 3. Discuss specific applications (e.g., identifying inbreeding depression loci, designing genetic corridors). Update IUCN citation (van Swaay et al. 2010 Latest assessment). 

 4. Define "Merian elements" upon first use (Page 5: "ancestral lepidopteran linkage groups"). 

 5. Provide ENA hyperlinks for raw data (e.g., HiFi: ERR13605515; Hi-C: ERR13602178).  Specify Ensembl annotation release timeline. 

 6. Use "kb" not "kilobases" (e.g., "15.51 kb"). Add commas to numbers (e.g., "1,836.76 ng" not "1 836.76 ng"). 

 7. Correct errors: "Bateman" (not "Baerman"); "Altschul" (not "Alicciato"); Nature (not "Not"). 

 8. Table 5: Replace "N/A" for *PretextSnapshot* with a version/git commit hash.

Are sufficient details of methods and materials provided to allow replication by others?

Yes

Is the rationale for creating the dataset(s) clearly described?

Yes

Are the datasets clearly presented in a useable and accessible format?

Yes

Are the protocols appropriate and is the work technically sound?

Yes

Reviewer Expertise:

Entomology, Bioinformatics

I confirm that I have read this submission and believe that I have an appropriate level of expertise to confirm that it is of an acceptable scientific standard.
